# Validation of questionnaire regarding online teaching (QOT) during Covid-19 in Karachi, Pakistan

**DOI:** 10.1371/journal.pone.0274268

**Published:** 2022-09-12

**Authors:** Najia Rahim, Shagufta Nesar, Tayyaba Mumtaz, Sadaf Naeem, Muhammad Ali

**Affiliations:** 1 Department of Pharmacy Practice, Faculty of Pharmaceutical Sciences, Dow University of Health Sciences, Karachi, Pakistan; 2 Jinnah College of Pharmacy, Sohail University, Karachi, Pakistan; 3 Department of Pharmacy Practice, Faculty of Pharmacy, Jinnah Sindh Medical University, Karachi, Pakistan; Universiti Malaya, MALAYSIA

## Abstract

The present study involved an extra-cultural adaptation and validation of questionnaire regarding online teaching (QOT) to know faculty perception, attitude and experiences of online teaching. Cronbach’s alpha was determined for assessing internal reliability of QOT and found to be 0.886, confirmed that the scale have good reliability. Factor Analysis of the scale (Principal Component Analysis) was used to examine factor structure and then trailed by varimax rotation. The items were allocated four sub scales. A survey technique was used for the validation of QOT and the survey was conducted during September-December’ 2020 in private and public universities of Karachi to determine the pharmacy faculty’s perception and experience regarding online teaching. Approximately 35% responded that “It is very easy to prepare and deliver an online course” and 45% opined that “The universities offering Pharm.D should adopt the use of e-learning for teaching in future to complement traditional teaching”. Majority of the faculty were in favor of starting online teaching during pandemic to complete semester on time (72%). However, in-campus courses contribute more to students’ learning than online courses in pharmaceutical sciences (65.5%). The present study summarizes that academic staff did opt online teaching over one-to-one teaching in the lock down situation during Covid-19 pandemic however, they agreed not to replace traditional teaching to online teaching. Teachers recognized some difficulties and challenges during online teaching including difficulty in preparing lecture for online teaching than traditional classroom teaching, shortage of long time training sessions. Female teachers were inclined to online teaching than male and lecturers tend to prefer online teaching compared to senior teachers. Universities and administration should take imperative acts for improving online teaching for better learning during lock down or any other situation where social distancing is required.

## Introduction

Online learning is described as learning through different Information and Communication Technologies (ICTs). Its growth is tremendous, more importantly within the context of higher education, primarily due to the tools used in online learning and the support given to students, teachers and their institutions. Keeping in view the economics of higher education, it is highly recommended that higher education institutions and students will continue to take an advantage of the online learning [1–3].

Covid-19 pandemic has become a global health issue, with about 90% of world population affected and it has significantly impacted education worldwide [4]. During Covid-19 lock down, traditional learning methods were modified to online learning. Academic institutions were under pressure to shoulder the responsibility. Academicians have to adapt to new online learning approaches. Although the shutdown of universities around the world due to the Covid-19 pandemic is unwelcome, it gives an immense opportunity for educational reform. To improve the overall learning environment, professional educators must incorporate blended learning into the curriculum and develop the best aspects of classroom and distant learning.

UNESCO shared recommendations for uninterrupted online learning for the lockdown period, however critics stated that online teaching is complicated due to the poor economic conditions in the middle-income countries [5]. Closure of educational centers not only affect learning but also affect the students’ assessments and examinations in developing countries like Pakistan, combined by a compromised educational system. Educators of such countries should have willingness to improve their systems. Additionally, the educational institutes are more responsible to develop secure, sound and economical online learning systems. Although this is difficult but can be made easy by spending time, make resources available for students and teachers to improve their educational system.

The sudden transition from traditional teaching to online teaching is a continuous process and faculties’, students’ and administrators’ experiences need to be assessed to identify challenges and strategies. Many studies conducted with the focus to gather students’ opinion and experiences of online teaching and learning in Covid-19 lockdown [6, 7] from Pakistan. Few studies were conducted to get faculty perception and experiences regarding online teaching [8, 9]. However, there is a deficiency in such studies that pharmacy faculty were not surveyed. Therefore, a questionnaire regarding online teaching was adopted, modified and validated to know faculty perception, attitude and experiences of online teaching in pharmacy education. Pharmacy education being a professional study is different from other school/colleges educations and no such report was published reporting Pakistani pharmacy academician response to online teaching. The focused group was teachers from pharmacy institutes/colleges however, this QOT can be used to get the faculty opinion involved in any other professional education.

## Methodology

In the present study, survey technique was adopted for the modification and validation of a questionnaire for evaluating the faculty response to online teaching including their perception, attitude and experiences [10–12]. Google forms was developed and used to get their response. During the lockdown period in 2020–21 due to Covid-19 crisis, different media outlets were used to distribute survey links via email, WhatsApp and messaging. The survey was conducted in the month of September-December’ 2020. The language of questionnaire was English.

### Ethical considerations

The ethical approval has been taken from Ethical Review Committee, Faculty of Pharmacy, Hamdard University to conduct the survey as google form (Ref.#. HU-ERC-20-230). Confidentiality of respondents’ information was maintained and verbal informed consent was taken before asking for the response to questionnaire.

### Sample size, inclusion/exclusion criteria

Raosoft^®^ online software was used to calculate sample size and for sampling non-probability snowball sampling technique was adopted. Approximately a total of 50 faculty members are present in different institutes of Karachi. Total population considered is 350 in pharmacy institutes (n = 7) whose first batch has been graduated. Margin of error was set 5% with 95% confidence interval. Sample size calculated was 184. Faculty was informed by head/chairman of the department to give response to questionnaire as Google Forms. After completion the questionnaires were later collected for data analysis. Faculty involved in under and post graduate pharmacy education were included in the survey. Unwilling participants were exempted from the study. The response rate observed in this analysis was 77%. The reason for low response was the online survey and teachers refused to respond due to their busy schedule when asked to respond to the questionnaire.

### Development and revalidation process of questionnaire regarding online teaching (QOT)

QOT questionnaire is based on five point Likert scale and its development and standardization involved different steps. It comprised of developing scale, reliability analysis and principal component analysis (PCA). The respondents can rate their responses on the basis of agreement or disagreement by choosing best appropriate responses (Likert 1932). The responses could be given as “strongly disagree”, “disagree”, “neutral”, “agree”, and “strongly agree”.

Factor analysis was used to determine the validity of the items in QOT, which enabled item scaling and assisted in determining the minimum number of dimensions required in the questionnaire in order to elucidate the maximum amount of information contained within the items and explain the correlation between them. Subscales were identified after doing an exploratory analysis with varimax rotation. The covariance matrix was used to conduct the research. The eigenvalues of factors with eigenvalues greater than 1.00 were assigned a number. The principal component analysis with varimax rotation was done afterward. All items were allocated to a factor with a loading greater than 0.45. The items were divided into four subscales using factor analysis. The Cronbach coefficient was used to assess the reliability of the data.

The first draft included 35 items was sent to ten experts from the field of pharmacy educational experts for screening. The draft was further improved and five items were deleted. Thirty items were retained in the final scale. At the last stage, the draft having 30 items was administered as Google form to different pharmacy colleges/institutes via email. The responses from the participants were gathered and analyzed by using SPSS 23 version.

### Statistical significant association analysis

An evaluation of the normality of data is a prerequisite for different statistical checks due to the fact that normality is an underlying assumption in many parametric testing. So, the sample was examined for normality before deciding the statistical test to be performed. Skewness and kurtosis were determined using SPSS 23 version. The data showed non-normal distribution, so the non-parametric tests were performed to analyze the influence of demographics of respondents on their responses. Clustering technique was adopted and such clusters were analyzed with Mann-Whitney’s U test (for two clusters i.e. age, nature of institute) and Kruskal-Wallis test (for >two clusters) for determining the statistical significant difference in their response between clusters.

## Results

### Characteristics of study population

Academician from pharmacy institutions (n = 184) were recruited through non probability sampling technique and asked to fill consent form. If they agreed, they were asked to respond to the questionnaire (QOT). A total of 142 academicians participated in the survey with the response rate of 77%. The characteristics of the study population are shown in Table 1. The respondents were also asked to express what content they uploaded online and the results are outlined in Table 2.

**Table 1:** Details of the pharmacy faculty participated in the survey (n = 142).

Position at University	N(%)
None	107(75.4)
Head of Section/Department	27(19.0)
Principal/Dean/Director	6(4.2)
**Academic Rank**	
Demonstrator/Instructor	13(9.2)
Lecturer	50(35.2)
Senior Academic Staff	79(55.6)
**Nature of University**	
Private	87(61.3)
Public	54(38.0)
**Sex**	
Female	101(71.1)
Male	40(28.2)
**Age**	
25–35 years	77(54.2)
36–45 years	44(31.0)
46–60 years	15(10.6)
Above 61	8(4.2)
**Working Experience**	
Less than one year	5(3.5)
Between two and five years	46(32.4)
Six to ten years	42(29.6)
Eleven years and above	49(34.5)
**Highest Academic Qualification**	
B.Pharm/Pharm.D	13(9.2)
Master /M.Phil	67(47.2)
PhD	61(43.0)
**Teaching Online Course**	
No	5(3.5)
Yes	134(94.4)
**Number of Course Taught Online/Semester**	
NIL	5(3.5)
1 to 2	72(50.7)
3 to 5	65(45.8)
**Having Knowledge on how to run online course**	
No	3(2.1)
Yes	136(95.8)
**Willing to teach online course**	
No	26(18.3)
Yes	114(80.3)

**Table 2:** Content type and techniques used by pharmacy faculty during online teaching.

**The content that you uploaded in E-learning/LMS Platforms**	**Yes N (%)**
Slides(Teaching Materials)	126(88.7)
Course Outline	127(89.4)
Journal Articles	71(50.0)
Course Introduction	122(85.9)
Books	124(87.3)
Learning Outcomes	113(79.6)
Study Cases	62(43.7)
Announcements	117(82.4)
Video Clip	75(52.8)
**The technique/tools that you used during online teaching**	**Yes N (%)**
Zoom	110(77.5)
Moodle	24(16.9)
Google classroom	73(51.4)
WhatsApp	94(66.2)
Skype	16(11.3)
Taking Quiz	108(76.1)
Marked Assignment	116(81.7)
Short Answers Questions	86(60.6)
Discussion Forum	104(73.2)

### Principal component analysis

Factor Analysis of the scale (Principal Component Factor) was used to examine factor structure and then trailed by varimax rotation. The suitability of data for execution of factor analysis was evaluated by Kaiser-Meyer-Olkin test and Bartlett’s test of Sphericity. The value of KMO test find out to be 0.837 (acceptable limits = value greater than 0.60). Bartlett’s test of Sphericity (Chi square: 2.136E3, p < 0.001) was found to be significant which also proved the validity of factor analysis and data was fit for reduction. Factor analysis was completed on 30 items. Four factors have eigen value higher than 1. Then parallel analysis was adopted in order to choose final number of factors for factor analysis and factor loading greater than 0.4 has been extracted. The eigen values for all the factors are 9.372, 2.481, 2.124 and 1.760. Total variance defined by each factor was found to be 31.247% (factor 1), 8.270% (factor 2), 7.080% (factor 3) and 5.867% (factor 4). These factors altogether, described 52.46% of the variance and these factors were provided names as per their characteristics of loaded items on the same factor.

**Fig 1:**
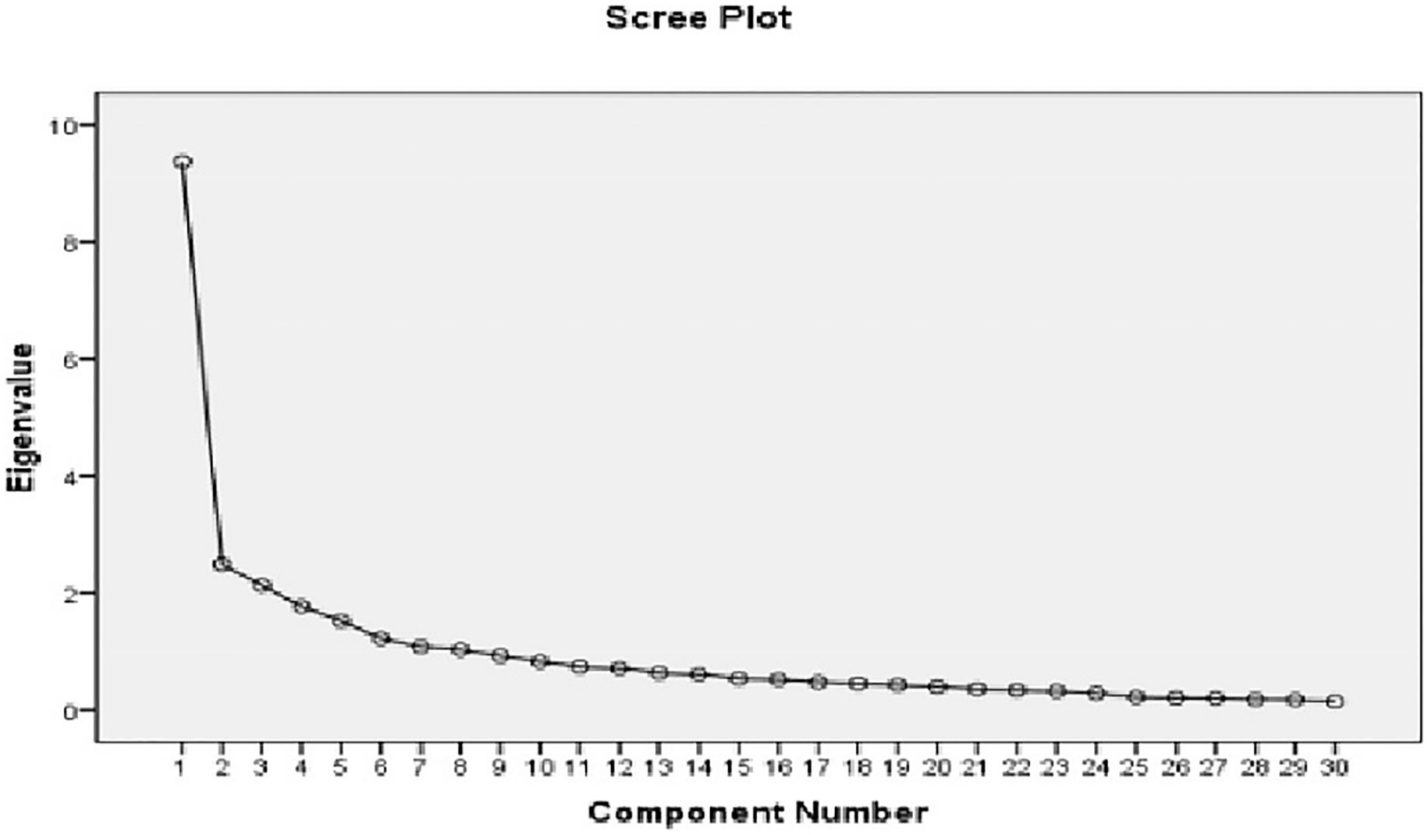
Scree plot of principal component analysis.

Fig 1 presented the Scree plot showing the curve with flattening after four factors; therefore, all the four factors for the scale can be retained. Then rotation was done and extracted factor are given in Table 3. The value of the factors ranged between 0.6 and 1 indicate high quality of factors loadings (Kline, 1994). The total of 30 items were qualified for the scale (Table 3). Out of total four factors, factors one includes 11 items (termed as “Acceptability”), factor 2 includes six items (i.e. Practicality), factor 3 includes nine items (termed as Technicality) and factor 4 includes four items (i.e. Discipline Specificity) mentioned in Table 3.

**Table 3:** Results of principal component analysis and pharmacy faculty responses.

Subscale	Items	Positive (%)	Mean	SD	Factor Loading
**Subscale 01 Acceptability**	Offering online courses will be very usefulness to University	53.6	3.46	0.90	0.614
	Shifting to online course will benefit my University	46.4	3.27	0.95	0.759
	Having online courses will have positive impact to our University	58.5	3.51	0.93	0.785
	There is an enabling environment in place to support the use of online courses	46.5	3.30	0.88	0.660
	If the university start offering online courses will be able to complete semester on time during pandemic	72.5	3.74	0.95	0.472
	The University popularity will increase if will offer online courses	54.2	3.40	0.96	0.800
	The universities offering Pharm.D should adopt the use of e-learning for teaching in future to complement traditional teaching	45	3.26	0.99	0.763
	Online courses will facilitate and assist my overall teaching	54.3	3.43	0.93	0.615
	Online courses will able me to plan better for my teaching	53.6	3.42	0.98	0.629
	Online courses will help to overcome the problem of a shortage of learning resources	62.8	3.55	0.91	0.746
	Online courses will make education more effective	42.2	3.13	1.01	0.627
**Subscale 02 Practicality**	It is very easy to prepare and deliver an online course	34.5	3.01	0.98	0.624
	We have e-learning experts who support me to prepare and deliver the course (s) at my University	39.4	3.18	0.98	0.626
	I have attended short training on how to prepare and deliver online courses	57.8	3.45	1.06	0.619
	I have attended long training to deliver online courses	21	2.59	1.06	0.643
	Lecturers are trained and have relevant and appropriate skills on online learning	55.6	3.42	0.89	0.543
	The academic staff has enough and relevant skills and knowledge to use online courses in teaching and learning	50.7	3.35	0.89	0.482
**Subscale 03 Technicality**	The IT infrastructure in my University support online course(s)	56.4	3.49	0.96	0.678
	The e-learning platform used by my University is of high quality	43	3.26	0.99	0.702
	There is enough e-learning facilities and equipment such as computers and laptops and Internet facilities	47.2	3.27	1.01	0.771
	There is stable Internet connection	35.2	2.98	1.07	0.696
	The ICT tools are constantly upgraded to keep them current	30.2	3.08	0.84	0.758
	There is standby power-generating to facilitate online courses at university campus	46.4	3.16	1.04	0.563
	We have enough Human Resources that is capable to prepare and deliver online courses at my University	43.6	3.14	0.97	0.559
	The University has sufficient financial resources to finance preparation and delivery of online courses	37.3	3.13	0.96	0.584
	There is an adequate fund for the institution to acquire the necessary online teaching facilities	38	3.15	1.012	0.755
**Subscale 04 Profession Specificity**	It need a lot of preparation to deliver online course(s)	31.7	3.43	1.06	0.483
	It need permission from Pharmacy Council of Pakistan to deliver online course(s)	55	2.97	0.95	0.572
	Traditional courses contribute more to students’ learning than online courses	65.5	3.78	0.90	0.536
	There is more difficult work involved to prepare online courses compared to traditional delivery of courses	59.2	3.56	0.97	0.693

### Reliability analysis

Cronbach’s alpha was determined for assessing internal reliability of the QOT and found to be 0.886, confirmed that the scale have good reliability (Cronbach 1951). The reliability of different subscales (n = 4) ranged between 0.656–0.902 which demonstrate the reasonable reliability of scale (Table 4).

**Table 4:** Results of internal reliability analysis.

Subscales of QOT	Description	Cronbach Alpha
Subscale 01	Acceptability	0.902
Subscale 02	Practicality	0.796
Subscale 03	Technicality	0.868
Subscale 04	Profession Specificity	0.656
**Total reliability score**		0.886

### Faculty responses and their significant association

Faculty perception, attitude and experiences of online teaching during Covid-19 was evaluated and mentioned in Table 3. Mean response to all items was found to be 3.32 with standard deviation of ±0.96. Their willingness to adopt online teaching was very encouraging (Subscale 01, mean 3.4, SD± 0.95). As far as their skills are concerned, in their opinion most of them have enough capability to continue online teaching especially in situations of pandemic like Covid-19 (Subscale 02, mean 3.17, SD ±0.98). However, technologically they face some inadequacy which in turn affect their teaching standard (Subscale 03, mean 3.18, SD±0.99). Being a professional health education, pharmacy faculty responded a mixed approach regarding online teaching pharmacy (Subscale 04, mean 3.44, SD±0.97). Gender, age, nature of university (public/private) and academic rank of faculty significantly influence their responses to different item outlined in Table 5.

**Table 5:** Statistical significant association of responses and demographic characteristics of pharmacy faculty.

Items in QOT	Nature of University	Gender	Age	Academic Rank	Highest Degree
If the university start offering online courses will be able to complete semester on time during pandemic	0.018*	0.033	0.025	0.001	---
I have attended short training on how to prepare and deliver online courses	<0.01	0.015	<0.001	0.011	---
Offering online courses will be very usefulness to University	---	0.039	---	<0.001	0.039
We have enough Human Resources that is capable to prepare and deliver online courses at my University	---	---	0.016	0.024	0.018
The e-learning platform used by my University is of high quality	---	---	0.003	---	0.04
Online courses will facilitate and assist my overall teaching	---	0.018	0.039	---	---
Online courses will able me to plan better for my teaching	---	---	0.045	0.04	---
It is very easy to prepare and deliver an online course	0.012	---	<0.001	---	---
The IT infrastructure in my University support online course(s)	---	---	---	---	0.009

*p<0.05 is considered significant, Mann-Whitney’s U test (for two clusters i.e. gender, nature of institute) and Kruskal-Wallis test (for >two clusters i.e. age, highest qualification, working experience, academic rank).

## Discussion

### Faculty response to subscale 01 i.e. acceptability

The present study revealed that faculty showed moderate acceptability to online teaching in Covid-19 scenario. They were inclined to have online teaching and learning in conjunction with traditional teaching to improve quality standards of teaching and learning. Faculty thought that online teaching would help them to improve their teaching (54.3%) and as well as beneficial to the university (46.4%). Faculty willingness to complete their courses on time is the main motive to adopt online teaching during pandemic. Pharmacy faculty from other parts of the world also quickly adopt the online teaching because of their vision to complete their teaching assignment within specified time duration without delaying semester which is unfavorable to students especially those who were graduating soon [13]. Study from Tanzania also reported that their faculty is highly inclined to adopt online teaching during Covid-19 [10]. Additionally other faculties from different universities also showed willingness to accept online teaching [14]. The academicians’ positive response assists in the implementation of the online learning in Covid-19 lockdown.

### Faculty response to subscale 02 i.e. practicality

Faculty recruited during the study responded that they have basic knowledge and skills to continue online teaching (approx. 50%). They get some short time training session by their universities (approx. 50%) however, they lack long term training program to improve their online teaching (approx. 20%). The lack of sufficient training of educators has been identified as a major obstruction to installing online learning [15]. A small number of faculty members showed unwillingness towards online teaching (18.3%). Another researcher from Pakistan also mentioned faculty members’ disinterest in adopting a new teaching technique, and sociocultural norms as an obstacle to online education [16]. In Pakistan, HEC closed institution and start online teaching without assessing preparedness of institutes and considering ground realities. However, later on they work on technological infrastructure development and faculty training [17].

### Faculty response to subscale 03 i.e. technicality

The rapid transition of classroom to online teaching raises some serious concerns. All though faculty showed acceptability for the adaptation, however they were very much concerned about technical facilities for both teachers as well as students for smooth delivery of online lectures. They mostly experienced the issue of unstable internet connection (58%). Availability of IT structure including equipment, internet in their university was ~60%. E-learning platforms used by different universities mainly depends upon their fund allocation for online teaching and learning. Human and financial resources available from the university is prerequisite for successful adaptation of online teaching. Faculty (38%) also complained the lack of funds available in university to continue online teaching and learning. However, they agreed that IT department of their university supports their online teaching (56.4%) and constantly upgrading their systems to meet the challenges (30.3%). Indonesian pharmacy teachers also opined that internet connectivity is the main obstacle in online teaching and learning [11]. The slow internet connectivity results in students’ disengagement during online teaching session [12]. Another report from Saudi Arabia also second the problems of technological, communication, students’ evaluation and health due to excess computer use [18]. Researchers from other cities of Pakistan also mentioned lack of internet connectivity, low bandwidth, frequent power failures as the obstacles to adopt new teaching strategies [16, 19]. It is indispensable that the institutions should invest in ICT infrastructure to improve the delivery of online courses.

### Faculty response to subscale 04 i.e. profession specificity

Pharmacy being a health care profession needs many skills to be learn by the students during their graduation studies. Online teaching of theoretical aspects is very effective and helpful to complete the courses during pandemic like Covid-19. However, practical and laboratory skills are efficiently learning during classroom education. Due to Covid restrictions, the strict protocols were applied by the hospitals, community health facilities, and pharmaceutical industries, the experiential teaching was sustained via online teaching. Major glitches of conducting laboratory practice and experiential learning online have also been reported in other countries, including Nigeria, Malta and Malaysia [13, 20, 21]. In pharmacy schools of Japan, the students require to complete a research project before graduation. In pandemic situation, projects involving hand-on participation in laboratories and clinical placements was restricted that resulted in lack of achieving learning outcomes [22]. Therefore, most of the pharmacy faculty responded in the survey agreed not to replace traditional to online teaching. Hybrid approach is a better choice in their opinion. Saudi pharmacy teachers were also in favor of hybrid approach especially in case of pharmacy education and suggested online teaching during any pandemic situation [12]. Another researcher also favors hybrid approach [13]. Online teaching has advantages in higher education but with continuing face to face module [23]. However, despite potential development of virtual learning for simulated experiences, for example using augmented and virtual reality, simulation cannot fully replace real clinical experiences [24]. Universities can implement hybrid strategies for coping the resource shortage, catering students from remote areas and interaction beyond one hour classroom lecture [20]. As Covid-19 lockdown restricts students from clinical placement and laboratory sites, pharmacy faculty strongly believed that virtual training cannot be efficiently train students as compare to real-life experimental training [25].

Pharmacy faculty also made efforts to prepare online lecture and other teaching material for students (31%) and faced difficulty in preparing online courses as compared to traditional teaching. Around 25% of pharmacy faculty globally felt difficulty in preparing online educational material and lecture content [25]. Case report from Nigeria showed that their pharmacy faculty experiences many challenges during online teaching in Covid-19 including infrastructural challenges, lack of appropriate training of academicians in ICT and lack of access to quality teaching resources [20]. Faculty from different pharmacy institutes also experience moderate level of work-related stress during online teaching in Covid-19 due to the factors like quickly adapting curriculum and assessments and rapid learning and usage of newer technologies [25]. University should respond to their faculty stress by providing reasonable work load and efficient IT infrastructure for online teaching. As in Pakistan like other developing countries majority universities do not have their e-learning platforms i.e. LMS (learning Management System). This Covid-19 led all the pharmacy institutions to upgrade their teaching technologies in conjunction with traditional mode and forcefully establishes a fixed system of e-learning in universities [18].

There are no precise guidelines dispensed for online health profession education neither by Pakistan Medical and Dental Council [19] or Pharmacy Council of Pakistan nor by Higher Education Commission of Pakistan for implementing online teaching and learning in Covid-19 situation [17]. However, medical, dental and pharmacy faculty and administrators, in the developing countries like Pakistan should try to convert the adversary of Covid-19 into an opportunity to develop e-learning programs. This is a learning experience for the faculty as well as the institutions that how to respond any pandemic in future.

### Significant association of faculty responses with their characteristics

Gender, age, nature of university (public/private) and academic rank of faculty significantly influence their responses to the different items of QOT (p<0.05). Females showed more positive response than male faculty. Study reported earlier from Pakistan mentioned that male university teachers were willing to teach online more than women [9]. However, Wang, et al., (2019) concluded that gender had no significant influence on online teaching [26]. But, the finding of the current study is consistent with many other studies reported that male university teachers used online teaching less [27, 28]. Working experience of faculty did not significantly influence their responses to all items of QOT. Other studies also confirms [29] that teaching experience motivate teachers for online teaching. Ph.D faculty gave more positive response about the availability different resources in their universities to facilitate their online teaching. Lecturers showed more positivity towards online teaching than senior faculty. However, another report from Pakistan mentioned that assistant professors are better than professors and lecturers in teaching online [9].

## Conclusion

This study summarizes that academic staff chose online over face-to-face teaching in the Covid19 pandemic lockdown, but agreed not to replace traditional teaching with online education. Teachers recognized some difficulties and challenges during online teaching including difficulty in preparing lecture for online teaching than traditional classroom teaching, shortage of long time training sessions. In contrast to other studies conducted during Covid-19 pandemic, this study found that lecturers tend to prefer online teaching compared to senior teachers. However, faculty needs continued support, training, development for this transition to online teaching. At the end of this global tragedy, we will be prepared to face any such situation in future without compromising professional education like pharmacy.

## Limitations and strengths

There is a wide scarcity of these types of validated survey tools to get faculty perception and attitude toward online teaching in any circumstances. Their responses and experiences give guidance to the institutions, accrediting bodies and government to continue teaching and learning in universities during pandemics. Although the survey tool was validated on pharmacy faculty, it can be used in other fields of education after revalidation. However, there are some limitations of this study, that we only get responses from institutes of Karachi so that results might not be generalized. Selection bias can result from sampling methods that is non-probability sampling technique, and it is not possible to see how representative the data are for the entire Pakistani population. Faculty refuses to answer the questionnaire, so a non-answer bias was also involved. Small sample size was also a limitation as the institutes where all five academic years were not enrolled, excluded from the study restricting the sample size. The results of the study are preliminary. One should be careful when using the current research results.

## Supporting information

S1 File (DOC)
