# Supplementary material for: Validation of questionnaire regarding online teaching (QOT) during Covid-19 in Karachi, Pakistan
Source: PLoS One. 2022 Sep 12;17(9):e0274268. doi: 10.1371/journal.pone.0274268 (PMC9467304; doi:10.1371/journal.pone.0274268)
Supplement: S1 File — (DOC) [file pone.0274268.s001.doc]

**Pharmacy Academicians’ Perception, Attitude and Experiences of Online Teaching during COVID 19 Pandemic**

**Background of Study:** The commonly methods used to teach courses in majority of Pakistani Universities is face-to-face mode. Covid 19 pandemic creates difficulty for most of universities to do it so. Therefore, on recommendations of Higher Education Commission of Pakistan most of the universities started Online Teaching in pharmacy colleges/faculties.

Thus, this study is planned to be conducted to determine the perception and experiences of pharmacy faculty member when they shift their teaching from face-to-face to online teaching.

**Demographic Details of Faculty Members**

| **Position at University** | |
| --- | --- |
| None |  |
| Head of Section/Department |  |
| Principal/Dean/Director |  |
| **Academic Rank** | |
| Demonstrator/Instructor |  |
| Lecturer |  |
| Senior Academic Staff |  |
| **Nature of University** | |
| Private |  |
| Public |  |
| **Sex** | |
| Female |  |
| Male |  |
| **Age** | |
| 25 -35 years |  |
| 36 - 45 years |  |
| 46 - 60 years |  |
| Above 61 |  |
| **Working Experience** | |
| Less than one year |  |
| Between two and five years |  |
| Six to ten years |  |
| Eleven years and above |  |
| **Highest Academic Qualification** | |
| B.Pharm/Pharm.D |  |
| Master /M.Phil |  |
| PhD |  |
| **Teaching Online Course** | |
| No |  |
| Yes |  |
| **Number of Course Taught Online** | |
| NIL |  |
| 1 – 2 |  |
| 3 - 5 |  |
| **Having Knowledge on how to run online course** | |
| No |  |
| Yes |  |
| **Willing to teach online course** | |
| No |  |
| Yes |  |

| **The content that you uploaded in E-learning/LMS Platforms** | **Yes** | **No** |
| --- | --- | --- |
| Slides(Teaching Materials |  |  |
| Course Outline |  |  |
| Journal Articles |  |  |
| Course Introduction |  |  |
| Books |  |  |
| Learning Outcomes |  |  |
| Study Cases |  |  |
| Announcement |  |  |
| Video Clip |  |  |
| **The technique/tools that you used during online** | **Yes** | **No** |
| Zoom |  |  |
| Moodle |  |  |
| Google classroom |  |  |
| WhatsApp |  |  |
| Skype |  |  |
| Taking Quiz |  |  |
| Marked Assignment |  |  |
| Short Answers Questions |  |  |
| Discussion Forum |  |  |

**Teachers’ Opinion and Experience of Online Teaching during COVID 19**

SA= strongly Agree, A=Agree, N=Neutral, D=Disagree, SD= Strongly Disagree.

| **Sr#** | **Items** | **SA** | **A** | **N** | **D** | **SD** |
| --- | --- | --- | --- | --- | --- | --- |
| 1 | It is very easy to prepare and deliver an online course |  |  |  |  |  |
| 2 | The IT infrastructure in my University support online course(s) |  |  |  |  |  |
| 3 | The e-learning platform used by my University is of high quality |  |  |  |  |  |
| 4 | We have e-learning experts who support me to prepare and deliver the course (s) at my University |  |  |  |  |  |
| 5 | I have attended short training on how to prepare and deliver online courses |  |  |  |  |  |
| 6 | I have attended long training to deliver online courses |  |  |  |  |  |
| 7 | There is enough e-learning facilities and equipment such as computers and laptops and Internet facilities |  |  |  |  |  |
| 8 | There is stable Internet connection |  |  |  |  |  |
| 9 | The ICT tools are constantly upgraded to keep them current |  |  |  |  |  |
| 10 | Lecturers are trained and have relevant and appropriate skills on online learning |  |  |  |  |  |
| 11 | There is standby power-generating to facilitate online courses at university campus |  |  |  |  |  |
| 12 | Offering online courses will be very usefulness to University |  |  |  |  |  |
| 13 | We have enough Human Resources that is capable to prepare and deliver online courses at my University |  |  |  |  |  |
| 14 | The University has sufficient financial resources to finance preparation and delivery of online courses |  |  |  |  |  |
| 15 | Shifting to online course will benefit my University |  |  |  |  |  |
| 16 | Having online courses will have positive impact to our University |  |  |  |  |  |
| 17 | There is an enabling environment in place to support the use of online courses |  |  |  |  |  |
| 18 | If the university start offering online courses will be able to complete semester on time during pandemic |  |  |  |  |  |
| 19 | The University popularity will increase if will offer online courses |  |  |  |  |  |
| 20 | It need a lot of preparation to deliver online course(s) |  |  |  |  |  |
| 21 | It need permission from Pharmacy Council of Pakistan to deliver online course(s) |  |  |  |  |  |
| 22 | The universities offering Pharm.D should adopt the use of e-learning for teaching in future to complement traditional teaching |  |  |  |  |  |
| 23 | Online courses will facilitate and assist my overall teaching |  |  |  |  |  |
| 24 | Online courses will able me to plan better for my teaching |  |  |  |  |  |
| 25 | The academic staff has enough and relevant skills and knowledge to use online courses in teaching and learning |  |  |  |  |  |
| 26 | Online courses will help to overcome the problem of a shortage of learning resources. |  |  |  |  |  |
| 27 | Online courses will make education more effective |  |  |  |  |  |
| 28 | There is an adequate fund for the institution to acquire the necessary online teaching facilities |  |  |  |  |  |
| 29 | Traditional courses contribute more to students’ learning than online courses. |  |  |  |  |  |
| 30 | There is more difficult work involved to prepare online courses compared to traditional delivery of courses. |  |  |  |  |  |
